# Foxa1 mediates eccrine sweat gland development through transcriptional regulation of Na-K-ATPase expression

**DOI:** 10.1590/1414-431X2022e12149

**Published:** 2022-08-15

**Authors:** Junhong Zhao, Lei Zhang, Lijie Du, Zixiu Chen, Yue Tang, Lijun Chen, Xiang Liu, Lei You, Yonghong Zhang, Xiaobing Fu, Haihong Li

**Affiliations:** 1 Department of Wound Repair and Dermatologic Surgery, Taihe Hospital, Hubei University of Medicine, Shiyan, Hubei, China; 2 Mental Health Center, Taihe Hospital, Hubei University of Medicine, Shiyan, Hubei, China; 3 School of Basic Medicine, Academy of Bio-Medicine Research, Hubei University of Medicine, Shiyan, Hubei, China; 4 Wound Healing and Cell Biology Laboratory, The First Affiliated Hospital, Chinese PLA General Hospital, Beijing, China; 5 Department of Plastic Surgery and Burn Center, The Second Affiliated Hospital, Shantou University Medical College, Shantou, Guangdong, China

**Keywords:** Forkhead box A1, Na-K-ATPase, Eccrine sweat glands

## Abstract

Eccrine sweat glands (ESGs) perform critical functions in temperature regulation in humans. Foxa1 plays an important role in ESG maturation and sweat secretion. Its molecular mechanism, however, remains unknown. This study investigated the expression of Foxa1 and Na-K-ATPase (NKA) in rat footpads at different development stages using immunofluorescence staining, qRT-PCR, and immunoblotting. Also, bioinformatics analysis and Foxa1 overexpression and silencing were employed to evaluate Foxa1 regulation of NKA. The results demonstrated that Foxa1 was consistently expressed during the late stages of ESGs and had a significant role in secretory coil maturation during sweat secretion. Furthermore, the mRNA abundance and protein expression of NKA had similar accumulation trends to those of Foxa1, confirming their underlying connections. Bioinformatics analysis revealed that Foxa1 may interact with these two proteins via binding to conserved motifs in their promoter regions. Foxa1 gain-of-function and loss-of-function experiments in Foxa1-modified cells demonstrated that the activities of NKA were dependent on the presence of Foxa1. Collectively, these data provided evidence that Foxa1 may influence ESG development through transcriptional regulation of NKA expression.

## Introduction

The skin is the largest organ in the human body and includes hair follicles, sebaceous glands, sweat glands, and other components. It protects internal organs and regulates body temperature. Eccrine sweat glands (ESGs) are found practically everywhere on the human skin and play a significant role in regulating body temperature, secreting up to 3-4 L of sweat every day (1,2). Sweat evaporation from the skin surface increases with hot conditions and strenuous physical labor (3). The function of ESGs is commonly lost as the result of congenital and acquired factors such as major burns or trauma of various etiologies, which can result in improper thermoregulation (4,5). Abnormal body temperature, particularly severe hyperthermia, can cause serious physical disability and even death (6,7). Structural damage and abnormal function of ESGs are detrimental to human survival and health; thus, physicians must determine how to repair and regenerate ESGs efficiently.

The Forkhead box A1 (Foxa1) protein belongs to a class of transcription factors known as pioneer factors (8). These pioneer factors bind to condensed, inactive chromatin and induce chromatin remodeling, making additional transcription factors in this area accessible (9,10). Foxa1 binds to its co-factors to stimulate the transcription of target genes (11). Foxa1 was shown to be highly expressed in late developing and developed mouse ESGs but not in early developing ESGs or hair follicles, and Foxa1 was linked to sweat secretion (12,13). Foxa1 knockout mice have integral ESG structures but loose sweat secretion function (12). Sweat gland base cells may express several ion channels, cotransporters, and enzymes such as Na^+^-K^+^-2Cl^-^ cotransporter 1 (NKCC1) and Na-K-ATPase (NKA) (14,15). These results suggest that these cells have a role in the reabsorption of certain ions during excretion. Sweat secretion will cease instantly following NKA blockade (1). NKA is found on the plasma membranes of nearly all eukaryotic cells (16). Na^+^ and K^+^ are transported across the membrane by the intracellular ATP hydrolase to create and maintain membrane potential, hence, directly and indirectly regulating certain key cell life activities (17,18). NKA can stimulate sweat secretion and ion exchange.

However, the expression and biological role of Foxa1 on NKA is yet to be determined (19,20). The aim of the present study was to uncover the detailed molecular functions of Foxa1 in rat ESG formation and analyze the interplay between Foxa1 and its candidate target NKA, thereby offering mechanical insight into how Foxa1 may mediate ESG development through transcriptional regulation of NKA gene expressions.

## Material and Methods

This study was submitted to and approved by the Research and Ethics Committee of Ethics in Animal Research of Shantou University Medical College (No. SUMC2017-018).

### Sampling rat footpads at different developmental stages

Adult Sprague Dawley rats (3 months old, weight 180-250 g) were purchased from the Beijing Vital River Laboratory Animal Technology Co., Ltd. (China). Female rats were mated with males, and the presence of a vaginal plug the following morning (day 0.5) indicated successful mating. Spontaneous delivery took place on day 21.5 of pregnancy. Footpads were collected from P1-P35 and 3 months at different development stages after birth, for subsequent hematoxylin-eosin (HE) staining, immunofluorescence staining, quantitative real-time reverse transcription-PCR (qRT-PCR), and immunoblotting. A subset of the samples was kept at -80°C.

### HE and immunofluorescence staining

Rat footpads were dissected in cold (4°C) 1× phosphate buffered saline (PBS) and fixed in 4% paraformaldehyde solution for 24 h at room temperature (24°C). Paraffin-embedded tissues were sectioned, deparaffinized, and hydrated. The HE staining kit (Beyotime, China) was used for the staining. Briefly, the sections (5-μm-thick) were stained with hematoxylin for 2 min and then immersed in the acidic liquid alcohol for 30 s. The images were obtained using an inverted microscope after staining with eosin for 2 min and dehydrated with ethanol (95, 100%). The samples were blocked with QuickBlock™ Blocking Buffer for Immunostaining (Beyotime) for 10 min, labelled with rabbit anti-Foxa1 antibody (Abcam, 1:400 dilution, UK), mouse monoclonal anti-NKA α2 (1:200 dilution, Santa Cruz, USA) for 8 h at 4°C, and then incubated with Alexa Fluor 546-labeled anti-rabbit IgG (H+L) secondary antibodies (1:500 dilution, Abcam) for 2 h in the dark. For counterstaining, the sections were incubated with 4′,6-diamidino-2-phenylindole (DAPI, Beyotime) in the dark for 1 min at room temperature. Finally, the sections were sealed with an anti-fluorescence quencher. The results were obtained using a Leica DMI8 fluorescence microscope (Leica, Germany).

### qRT-PCR

RNA was isolated from footpad tissue of rats at different developmental stages as well as from HEK293T/Hela cells with an RNA extraction kit (Takara, Japan). From each sample, 1 µg of RNA was used to synthesize cDNA with the SuperScript III First-Strand Synthesis Supermix (Vazyme, China). qRT-PCR analysis of Foxa1 NKA was performed on the Applied Biosystems Quant Studio system (Life Technologies, USA) using 2× ChamQ Universal SYRB Green qPCR Master Mix (Vazyme). The total reaction volume was 20 µL, including 10 µL 2× ChamQ Universal SYRB green qPCR Master Mix (Vazyme), 0.8 µL of reverse and forward primers (10 µM), 7.8 µL of DEPC water, and 1 µL of cDNA (100 ng/µL). The gene expression of Foxa1 NKA was studied in three biological replicates, and the data for each sample were standardized to the levels of glyceraldehyde-3-phosphate dehydrogenase (GAPDH) expression using the 2^-ΔΔCT^ method. The primers are listed in Table 1.

**Table 1:** Primer sequences.

Primer name	Primer sequence (5′-3′)
Human-Foxa1-F	TCCGCCACTCGCTGTCCTTC
Human-Foxa1-R	CGCAAGTAGCAGCCGTTCTCG
Human-GAPDH-F	GAGTCAACGGATTTGGTCGT
Human-GAPDH-R	TTGATTTTGGAGGGATCTCG
Human-NKA α1-R	ATCGTTTTGAGGTTCCTCTTCT
Human-NKA α1-F	ATCTTCCTCATCGGCATCATAG
Human-NKA α2-F	ATCTTCCTCATCGGCATCATAG
Human-NKA α2-R	CTCATGGATTTGGTTGTCGAAC
Human-NKA α3-F	GACCTCATTTGACAAGAGTTCG
Human-NKA α3-R	ATTTGTTGGTGGAATTGAAGGG
Human-NKA β1-F	TAAAGCTCAACCGAGTTCTAGG
Human-NKA β1-R	TGGGTTATACTTCATCACTGGG
Human-NKA β2-F	CAGAGCATGAATGTTACCTGTG
Human-NKA β2-R	TAGTAGGGGAAGTACATGAGGT
Human-NKA β3-F	TGATGGAGCACTTTTTGAACAG
Human-NKA β3-R	ACTCCTTCAGGCTTTAATCCAA
Rat-Foxa1-F	CGCCCTACTCCTACATCTCG
Rat-Foxa1-R	GCTGCTGGTTCTGACGGTAAT
Rat-GAPDH-F	CAGTGCCAGCCTCGTCTCAT
Rat-GAPDH-R	AGGGGCCATCCACAGTCTTC
Rat-NKA-F	GCTCTTGCTGCTTTCCTGTCCTAC
Rat-NKA-R	GCTTCCGCACCTCGTCATACAC
Foxa1-cds-F	tccgaattcatcgatggccggccGATGTTAGGAACTGTGAAGATGGAA
Foxa1-cds-F	catgtgcagtagtgaggcgcgccCTAGGAAGTGTTTAGGACGGGTCT

### Immunoblotting

Total protein was recovered from rat footpad tissues at different developmental stages and HEK293T/Hela cells using RIPA lysis buffer (Beyotime). Samples were separated by 10% SDS-PAGE electrophoresis and transferred to PVDF filters. Immunoblottings were probed with rabbit monoclonal anti-Foxa1 (Abcam, 1:2000 dilution), mouse monoclonal anti-NKA α2 (Santa Cruz, 1:3000 dilution), and rabbit monoclonal anti-GAPDH (Abcam, 1:5000 dilution) antibodies for 2 h at room temperature. Following three 10-min washes in TBST buffer (10 mM Tris-HCl (pH 7.4), 300 mM NaCl, and 0.1% (v/v) TWEEN20), membranes were incubated with either the HRP-conjugated anti-mouse IgG (Beyotime, 1:2000 dilution) or the HRP-conjugated anti-rabbit IgG (Beyotime, 1:2000 dilution) at room temperature for 1 h. The membranes were then subjected to another three 10-min washes in TBST buffer and developed. Finally, the signal was detected using an enhanced chemiluminescence reagent (Millipore, USA) and recorded with the ChemiDoc Touch Imaging System (Bio-Rad, USA).

### Iodine sweat test

Rats were given 500 µL of sodium pentobarbital and the plantar surface of a rear paw was coated with iodine/alcohol (2 g iodine/100 mL ethanol) for the iodine sweat test. A starch-oil suspension (2 g starch/2 mL castor oil) was applied to the paw surface after it dried. Fine black dots revealed functional ESGs pores.

### Construction and transfection of Foxa1 recombinant plasmid

Full-length cDNA encoding human Foxa1 was constructed on pCS2-EGFP vectors donated by FJNU Biomedical Research Centre of South China (China) through DNA recombination technology. To create the recombinant plasmid pCS2-EGFP-Foxa1, the cDNA and pCS2-EGFP vectors were digested with FseI and AscI, ligated together, and transformed into *E. coli* Top10 competent cells. DNA sequencing revealed accurately constructed plasmids. The primers and sequences are listed in Table 1. The HEK293T and Hela cells purchased from the Cell Bank of the Chinese Academy of Science were cultured in DMEM with 10% fetal bovine serum (FBS) and 1% penicillin/streptomycin and maintained at 37°C in a humidified incubator with 5% CO_2_. All cell lines were validated and mycoplasma-free tested.

Cell transfection was performed in a 6-well plate with cells grown to 85% density using Lipofectamine 2000 transfection reagent (Life Technologies, USA). HEK293T and Hela cells were collected 24 h post-transfection to detect the expression of Foxa1 and NKA.

### Synthesis and transfection of Foxa1 siRNA

Foxa1-siRNA, negative control (NC) siRNA, positive GAPDH control siRNA, and fluorescent (FAM) siRNAs were designed and synthesized by the Tsingke Biological Technology (China). The siRNA and sequences are listed in Table 2. HEK293T and Hela cells were plated in 6-well plates at 60% confluence and then transfected with Foxa1 siRNA (50 nM) using Lipofectamine 2000 transfection reagent, according to the manufacturer’s protocol. The cells were collected 48 h after transfection for fluorescence examination, immunoblotting, and qRT-PCR analysis.

**Table 2:** SiRNA targeting sequences for human Foxa1.

Gene	Human Foxa1 siRNA sequence
Foxa1 siRNA 1	Sense 5′-GCGACUGGAACAGCUACUATT-3′
Foxa1 siRNA 1	Anti-Sense 5′-UAGUAGCUGUUCCAGUCGCTT-3′
Foxa1 siRNA 2	Sense 5′-CCACUCGCUGUCCUUCAAUTT-3′
Foxa1 siRNA 2	Anti-Sense 5′-AUUGAAGGACAGCGAGUGGTT-3′
Foxa1 siRNA 3	Sense 5′-GCACUGCAAUACUCGCCUUTT-3′
Foxa1 siRNA 3	Anti-Sense 5′-AAGGCGAGUAUUGCAGUGCTT-3′

### Immunofluorescence analysis to confirm Foxa1 protein expression

Immunofluorescence analysis was performed as described previously in Material and Methods. The cell slides were sterilized by immersion in 70% alcohol for 30 min. HEK293T and Hela cells were seeded in a 24-well plate with a cell slide at a density of 1×10^4^ cells per well. The cell slides were removed after 24 h of transfection. Finally, the expression and localization of Foxa1 in cells were examined by immunofluorescent staining. Cell slides were treated in PBS containing 5% bovine serum albumin (BSA) and 0.5% Triton X-100 for 30 min to inhibit non-specific binding, followed by a 2-h incubation with rabbit anti-Foxa1 antibody (Abcam, 1:400 dilution) at room temperature. After three PBS washes, the slides were incubated with Alexa Fluor546-labeled anti-rabbit IgG (H+L) secondary antibody (Abcam, 1:500 dilution) in the dark for 2 h. The results were obtained using a Leica DMI8 fluorescence microscope (Leica).

### Statistical analysis

Data were analyzed using GraphPad Prism 8.0 software (GraphPad Software Inc., USA). Variance comparisons were performed using one-way ANOVA, with P<0.05 and P<0.01 deemed statistically significant. The results are reported as means±SE.

## Results

### Expression pattern of Foxa1 during ESG development

**Figure 1:**
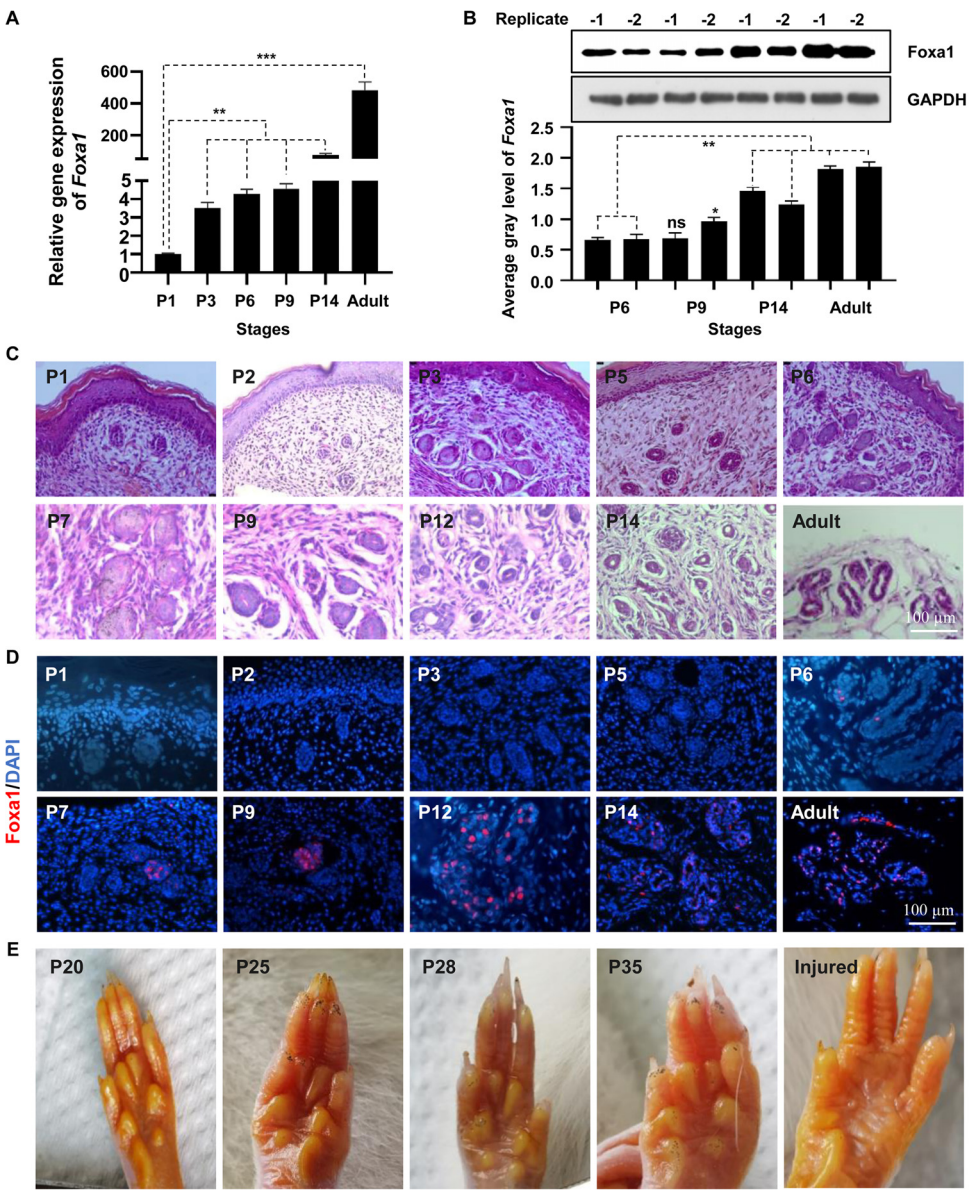
Expression pattern of Foxa1 during rat eccrine sweat glands (ESG) development. **A**, Data are reported as means±SE. qRT-PCR analysis of Foxa1 mRNA expression during different development stages of postnatal rat footpads. **B**, Immunoblotting showing Foxa1 expression during rat ESG development. The average grey levels were calculated using ImageJ (NIH, USA). **C**, HE staining of ESGs at different development stages of postnatal rat footpads (20×, scale bar: 100 µm). **D**, Immunofluorescence staining showing Foxa1 expression in postnatal rat footpads at different development stages (20×, scale bar: 100 µm). **E**, Iodine-starch detection of sweat secretion in the rat footpads at different development stages and the lost metatarsal footpads (injured footpads). **A** and **B**: Data are based on n=5 for each group. *P<0.05; **P<0.01; ***P<0.001; ns: not significant (ANOVA). P1 to 35: number of postnatal days and adult (3 months old).

Foxa1 is a conserved transcriptional factor that is expressed in various tissues and organisms. To investigate the molecular characteristics of Foxa1, we first tracked its expression patterns in rat footpad tissue beginning on postnatal day 1 (P1), when the secretory coil (SC) starts to develop (21). We discovered that transcriptional activity increased drastically from P3 and remained stable into adulthood (Figure 1A). Immunoblotting confirmed that Foxa1 protein accumulated in rats with the formation of ESGs (Figure 1B). To back up these findings, we used histological sections and HE staining to track the precise developmental stages of ESGs, and the results revealed that SC emerged in P1 (Figure 1C). Moreover, immunofluorescence data demonstrated that the Foxa1 fluorescence signal could be detected as early as P6 and peaked at P12 (Figure 1D). Intriguingly, we discovered a strong iodine-starch staining, which indicated intense sweat secretion (Figure 1E). Overall, these data not only provided an efficacious examination of Foxa1 expression patterns in ESG development but also demonstrated its significance in sweat secretion.

### Increased NKA expression during rat ESG development

**Figure 2:**
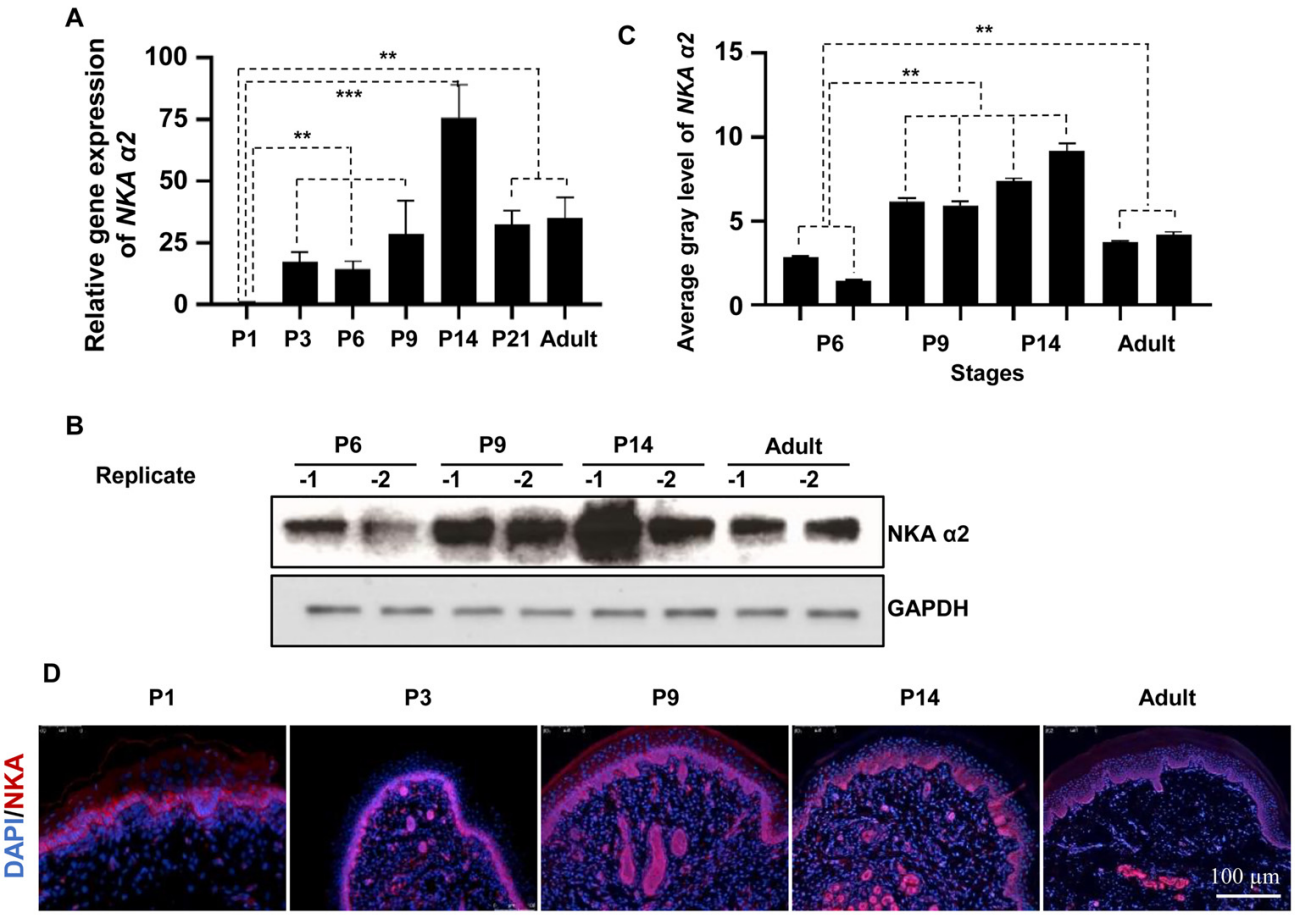
NKA expression increases during eccrine sweat gland development. **A**, qRT-PCR analysis of NKA mRNA expression in rat footpads during different postnatal development stages. **B** and **C**, Immunoblotting showing protein expression levels of NKA during rat ESG development. The average grey levels were calculated using ImageJ (NIH, USA). **D**, Immunofluorescence staining showing NKA expression in rat footpads at different postnatal development stages (20×, scale bar: 100 µm). **A**-**C**, Data are reported as means±SE; n=5 for each group. **P<0.01; ***P<0.001, ANOVA. P1 to 21: number of postnatal days and adult (3 months old).

A recent investigation in our laboratory based on immunofluorescent detection of the expression patterns of Foxa1 and NKA found that they are colocalized in three-dimensional (3D) reconstructed human ESGs, the functional counterpart of native ESGs (21); these data indicated that Foxa1 and NKA may play a role in ESG formation. In the present investigation, we sought to reveal whether and how these two components interact in rat ESG development. qRT-PCR assays revealed that the mRNA abundance of NKA increased progressively during the postnatal development of rat ESGs in a manner comparable to Foxa1 (Figures 1A and 2A-C). As expected, protein expression levels of Foxa1 and NKA increased accordingly. Moreover, immunofluorescence data demonstrated that the NKA fluorescence signal could be detected from P1 and peaked at P14 (Figure 2D). These temporal expression differences supported the notion that these three components might interact to mediate ESG formation.

### Foxa1 potentially mediated ESG development by promoting transcriptional activities of NKA

**Figure 3:**
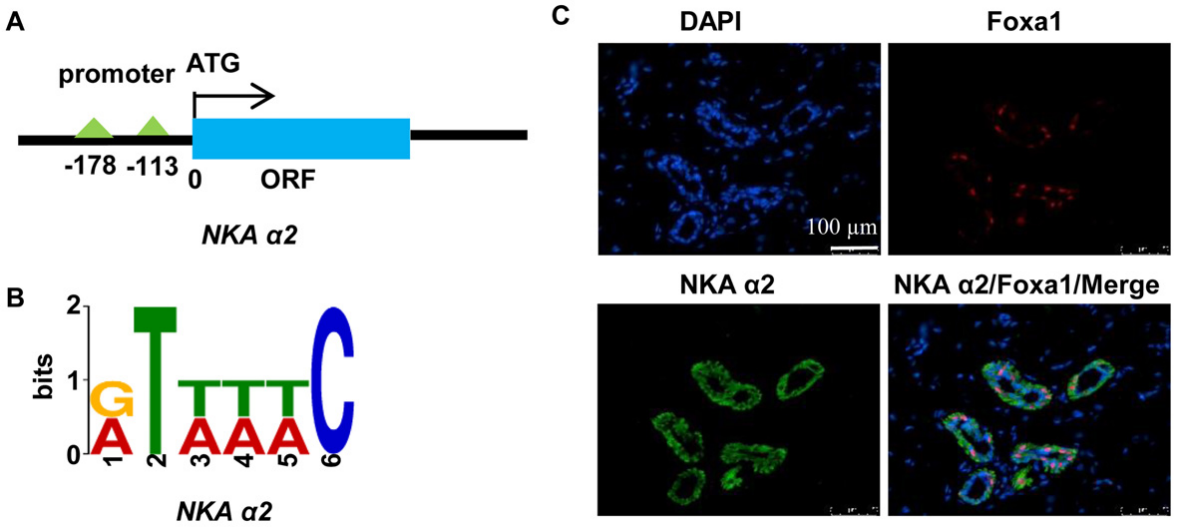
Predicted Foxa1 conserved binding motif at promoter regions of NKA. **A**, Gene models. **B**, Conserved DNA sequences in NKA. **C**, Immunofluorescence staining showing Foxa1 and NKA expression in rat footpads. Scale bar: 100 µm.

Previous evidence indicates that Foxa1 acts as a transcription factor, directly binding to a conserved cis-element, 5′-[AC]A[AT]T[AG]TT[GT][AG][CT]T[CT]-3′, to regulate the expression activity of its downstream targets (22). Given the findings presented above, we hypothesized that Foxa1 may bind to NKA and regulate its transcriptional activity. As such, we used the PROMO database (http://alggen.lsi.upc.es/cgi-bin/promo) to predict the Foxa1 binding motif. Fortunately, one and two conserved Foxa1 motifs were discovered in the proximal regions of NKA promoters (Figure 3A and B). In addition, immunofluorescence showed that endogenous Foxa1 and NKA co-localized in rat footpads. These results suggested a likely interaction between Foxa1 and NKA (Figure 3C). Thus, Foxa1 is thought to directly bind and regulate NKA.

**Figure 4:**
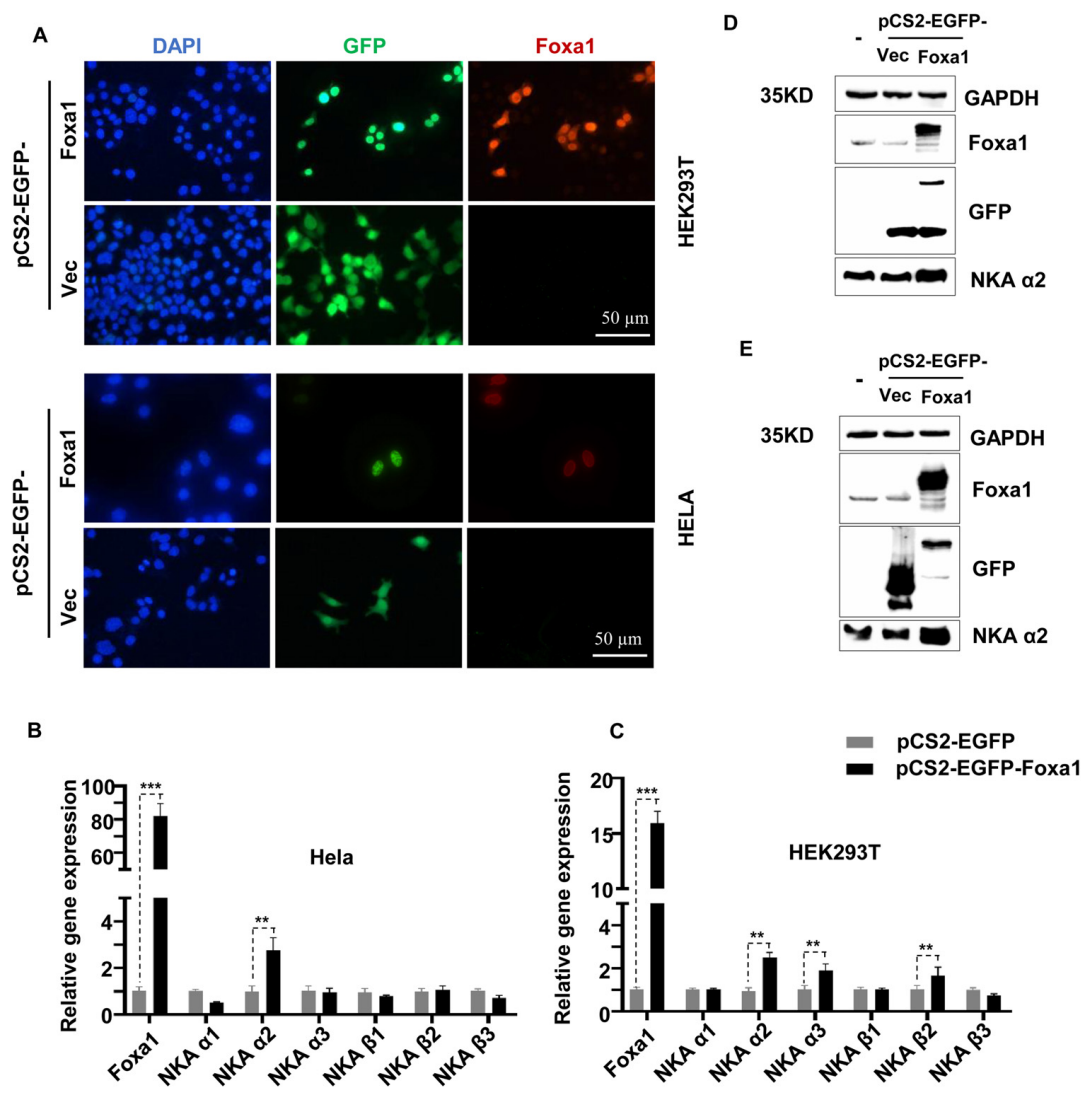
Increased expression activities of NKA in Foxa1 overexpression cell lines. **A**, Immunofluorescence analysis of the subcellular localization of Foxa1. Blue, DAPI; green, GFP; red, Foxa1. (40×; scale bar: 50 µm). **B** and **C**, qRT-PCR analysis of mRNA levels of NKA subunits in Hela and HEK293T cells overexpressing Foxa1. Data are reported as means±SE. **P<0.01; ***P<0.001 (ANOVA). **D** and **E**, Immunoblotting showing protein expression levels of NKA in HEK293T and Hela cells overexpressing Foxa1.

**Figure 5:**
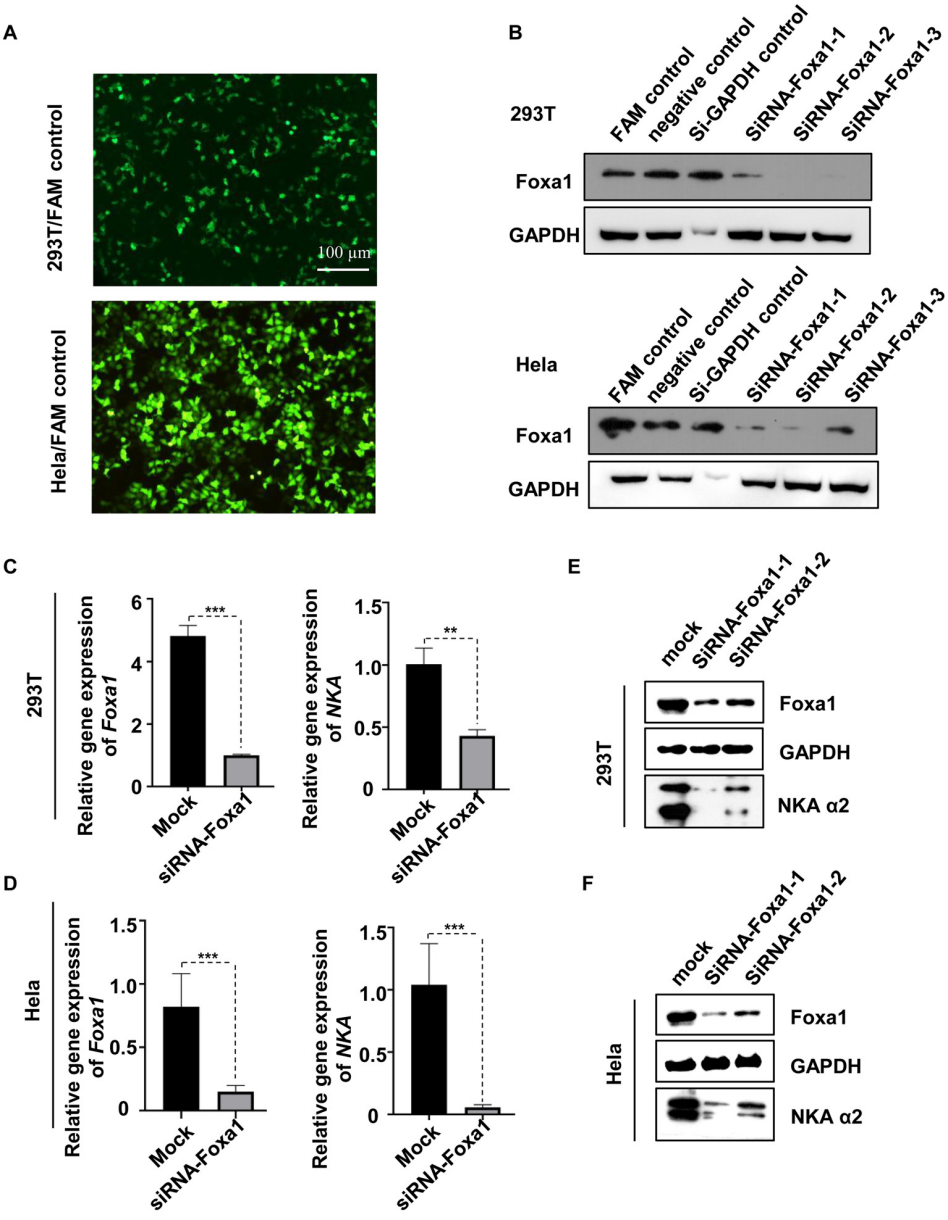
Decreased expression activities of NKA in Foxa1 knock-down cell lines. **A**, Immunofluorescence analysis of the subcellular localization of siRNA transfection effects (10×; scale bar: 100 µm). **B**, Immunoblotting assay showing Foxa1 protein level with Foxa1-siRNA treatment. **C** and **D**, qRT-PCR analysis of mRNA levels of NKA subunits in HEK293T and Hela cells overexpressing Foxa1. Data are reported as means±SE. **P<0.01; ***P<0.001 (*t*-test). **E** and **F**, Immunoblotting results showing protein expression levels of NKA in HEK293T and Hela cells overexpressing Foxa1. FAM: fluorescent.

Furthermore, Foxa1 gain-of-function and loss-of-function genetically engineered cell lines were constructed to verify and acquire molecular insights into the relationship between Foxa1 and the two transporter encoding genes. The constructs were introduced in two of the most regularly used cells, HEK293T and Hela, to strengthen our prediction. Compared to the empty construct (Vec), we found higher expression levels of Foxa1 in pCS2-EGFP-Foxa1, the gain-of-function cell lines (Figure 4A). These data were further supported by qRT-PCR and immunoblotting results (Figure 4B-E). The qRT-PCR analysis revealed that increased Foxa1 expression resulted in elevated amounts of NKA, particularly NKA α2 (Figure 4B and C). The immunoblotting results were consistent with the gene expression detection (Figure 4D and E), demonstrating that Foxa1 overexpression increased NKA protein levels. Meanwhile, using the siRNA method, we successfully created Foxa1 loss-of-function (Figure 5). The two Foxa1 knock-down cell lines exhibited lower NKA transcriptional and protein levels compared with their negative counterpart. Overall, the genetic data suggested that Foxa1 may play a role in ESG formation by positively regulating NKA transcriptional activity.

## Discussion

Evidence from this study revealed elevated Foxa1 expression during ESG development. It was notable that NKA expression steadily increased and peaked at 14 days after birth. These findings demonstrated a potential correlation between NKA and Foxa1, while it was not synchronized. In addition, Foxa1 was found to play a positive role in regulating the growth and maturation of ESGs in the context of Foxa1 knockdown in mammalian eukaryotic cells. The expression of NKA decreased following the Foxa1 knockdown. These data supported the notion that Foxa1 regulated the expression of the NKA α2 subunit.

As a key organ, ESGs perform the primary cooling function when the human body is subjected to a heated environment, physical exercise, or fever (23). The abnormal development of ESGs can cause anhidrosis, hyperhidrosis, or hyperhidrosis, all of which significantly affect human life (24,25). Cui et al. (12) showed that the Forkhead transcription factor, Foxa1, is required for mouse sweating capacity. In addition, sweat glands of *Foxa1* gene knockout mice lose their sweating function. In our previous investigation, Foxa1 was revealed to play a role in the 3D reconstructed ESGs *in vitro*, and Foxa1 expression increased with *in vitro* culture time (13). However, the link between Foxa1 expression and sweat functional protein Na-K-ATPase is not completely understood.

There are several sodium-potassium pumps in the folds created by the cell membrane in the secretory region of the human exocrine sweat glands, which supply the driving force for the sodium-potassium-chloride transporter (26-28). Emerging evidence shows that the Na^+^/K^+^-ATPase marker is one of the sweat gland-specific markers (29,30). Indeed, Na^+^/K^+^-ATPase has been shown to modulate human sweating during exercise; this is evidenced by the dramatic decrease in sweat rate following topical administration of the Na^+^/K^+^-ATPase inhibitor ouabain (31,32). Louie et al. (33) found that Na-K-ATPase is implicated in the regulation of the cutaneous vasodilatory response and sweating during moderate- and high-intensity exercise. NKA is an ion transporter, and inhibiting Na-K-ATPase may interrupt sweat secretion (24,34).

In keeping with this line of research, we found that Foxa1 influenced NKA expression in rat ESGs. These early findings indicated that Foxa1 regulated mRNA and protein expression of the Na-K-ATPase α2 subunit but not the α1 and α3 subunits. The alpha and beta subunits of the Na-K are the most important. The α subunit is divided into three subunits, α1, α2, and α3, which include binding sites for Na and K ions as well as ATPase activity that potentially catalyzes the hydrolysis of ATP (35). The α subunit is the “housekeeping” subunit, and the β subunit is a small subunit, a glycoprotein with a molecular weight of about 50 kD, that primarily regulates the number of sodium pumps released to the plasma membrane by assembling α/β dimers (16,36). Na-K-ATPase α2 catalyzes the hydrolysis of ATP and the exchange of sodium and potassium ions across the plasma membrane (37-39). An electrochemical gradient of sodium and potassium is created as a result of this activity, providing energy for active nutrient transport.

We looked into the more important role of Na-K-ATPase α2 in sweat secretion and material exchange. Despite multiple investigations on the expression and function of Na-K-ATP in sweat glands, no studies have demonstrated that the α2 subunit plays a critical role in sweat gland development and sweat secretion. We found that Foxa1 regulated the expression of gene Na-K-ATPase α2, and these findings have implications for understanding sweat gland development and function. We now understand that NKA is much more than a pump. It functions as a receptor for both endogenous and exogenous ligands, activating different signaling pathways associated with cell survival, differentiation, proliferation, and apoptosis among other things. Although Luo and colleagues showed that the small membrane protein FXYD6 could increase NKA expression, whether it can improve sweat gland function remains to be determined (40).

In future research, we will attempt to elucidate the interaction between Foxa1 and NKA in 3D reconstructed glands or human ESG tissue. Furthermore, it is difficult to investigate the role of Foxa1 regulation of NKA α2 in the sweat gland because of its complex nature and the challenges in dealing with and transfecting primary sweat gland cell cultures. Also, we will investigate the effect of knocking out Na-K-ATPase α2 subunits on the developmental morphology and function of sweat glands in mice and whether Foxa1 plays a regulatory role in this process.

As previously stated, sweat secretion-related proteins are known to influence the development of ESGs; however, a notable finding of the present study was that transcription factor Foxa1, a critical signaling mediator, can act on these sweat secretion-related proteins and influence the ability of ESGs to perform their normal functions.
